# Mixed Infant Feeding Practice and Associated Factors among HIV-Positive Women under Care in Gondar City's Public Health Facilities within Two Years Postpartum: A Cross-Sectional Study

**DOI:** 10.1155/2020/4597962

**Published:** 2020-06-20

**Authors:** Muhabaw Shumye Mihret, Mengstu Melkamu Asaye, Banchigizie Adane Mengistu, Habte Belete

**Affiliations:** ^1^Department of Clinical Midwifery, School of Midwifery, College of Medicine and Health Sciences, University of Gondar, Ethiopia; ^2^Department of Women's and Family Health, School of Midwifery, College of Medicine and Health Sciences, University of Gondar, Ethiopia; ^3^Department of Midwifery, Teda Health Sciences College, Amhara Regional Health Bureau, Ethiopia; ^4^Psychiatry Department, College of Medicine and Health Sciences, Bahir Dar University, Bahir Dar, Ethiopia

## Abstract

**Background:**

Mixed infant feeding practice remains a major setback for effective prevention of mother to child transmission of HIV program and updated evidences on this issue is essential for better interventions. Therefore, this study was aimed at assessing the proportion and associated factors of mixed infant feeding practice among HIV-positive women under care in public health institutions in Gondar city within two years postpartum, Ethiopia, 2017.

**Methods:**

A cross-sectional study was conducted on 485 HIV-positive women under care in Gondar City's health facilities from May 1 to June 30/2017. Data were collected via interviewer administered questionnaire supplemented with chart review, entered into Epinfo version 7.0 and then exported to SPSS version 20.0. Both bivariable and multivariable analyses were done, and the statistical significance of each variable was claimed based on the adjusted odds ratio (AOR) with 95% confidence interval (CI) and its *P* value ≤0.05.

**Result:**

The proportion of HIV-positive women practicing mixed infant feeding was 21.6%. Whereas, about 73.8% and 4.5% of the mothers demonstrated exclusive breastfeeding and exclusive replacement feeding, respectively. Mixed infant feeding practice was independently predicted by lack of antenatal care (AOR = 6.9; 95% CI: 3.4, 14.1) and home delivery (AOR = 2.8; 95% CI: 1.4, 5.4).

**Conclusion:**

The magnitude of mixed infant feeding practice was higher than the reports of many other studies, and its predictors were connected to poor adherence to maternal health care service utilization. Hence, stakeholders need to work more on ANC and facility delivery service coverage.

## 1. Introduction

More than 90% of the HIV-infected children and nearly 88% of the newly infected children are living in Sub-Saharan Africa (SSA) [1]. As a part of SSA country, Ethiopia is also largely attacked by the burden. Researches depict that the main cause of under 5 years children HIV infection is vertical transmission of the virus from mother to child [2]. In relation to vertical transmission of HIV, infant feeding practice plays a compulsory role [3–5].

Infant feeding practice can be categorized into recommended (i.e., either exclusive breast or replacement feeding) and nonrecommended (i.e., mixed feeding) practice. Generally, either exclusive breastfeeding (EBF) or exclusive replacement feeding (ERF) is advisable over mixed infant feeding (MIF) during the first 6 months of child age [6–8]. HIV-infected mothers can practice ERF only when replacement feeding is affordable, feasible, acceptable, sustainable, and safe (AFASS). However, the majority of the mothers living in resource-limited countries cannot fulfill the AFASS criteria. Therefore, EBF accompanied by adherence to HIV treatment gives HIV-exposed infants the best chance to survive and thrive HIV-free in settings where AFASS are not guaranteed and child deaths from undernutrition, pneumonia, and diarrhea are widespread [9]. EBF is also associated with a reduced risk of vertical transmission of HIV when compared to MBF in the early months of postpartum [10]. In spite of this fact, the rate of EBF practice is not satisfactory and varied across settings due to enormous barriers and determinants globally—being lower in SSA including Ethiopia [11].

The practice of EBF is a general recommendation for all mothers disregard to their HIV status [4]. However, more strict emphasis is provided for HIV-positive mothers, since MIF practice imposes vertical mother to child transmission (MTCT) of HIV along with many other adverse outcomes, and thus, the prevalence of EBF practice is expected to be more common in HIV-positive women [5]. In Ethiopia, the pooled prevalence of EBF practice is 59.3% in the general population [12] and 63.4% among HIV-positive women [13] which is comparable to the figures in most SSA countries, but, lower compared with the global figure and inconsistent to the strategies intended to eliminate MTCT of HIV by 2030 [5, 11]. On the other hand, the practice of MIF in the first six months of infant age remains high (23.1%) in Ethiopia [13]. MIF practice is also common in the study area—Gondar city. A recent descriptive cross-sectional study done in Gondar city among 402 HIV-positive women reported that about 76.3% of them had poor infant feeding practice. This despicable practice could be due to poor knowledge and unfavorable attitude towards infant feeding practice as evidenced by: 56.0% of them believed that MIF has no a risk of HIV infection, 73.9% of the women thought that EBF is not good since it transmits HIV, and 63.5% of them agreed that they do not accept EBF for fear of stigma due to HIV [14]. In this regard, evidences showcase that noticeable vertical transmission is undertaken through malpractice in breastfeeding [15, 16]. Therefore, MIF practice in the first six child's age is a very serious issue and totally unacceptable due to its great part in MTCT of HIV and other adverse health outcomes [6–10, 17–19].

In light of global and country commitments to the elimination of new pediatric infections in relation to target three of Sustainable Development Goal (SDG 3) [20], all countries have been pushed to apply evidence-based effective strategies of prevention of mother to child transmission (PMTCT) of HIV [2, 7, 8, 15, 21]. Since MIF practice is a major setback for effective PMTCT program, and updated information is the blueprint for the program and policy makers, the necessity of continuous investigation on MIF practice is essential. In connection to this, there was a dearth of studies more particularly in the study area, and even the existing studies are either too old (i.e., had been done 5 years back) [16] or descriptive in nature [14]. Moreover, evidences could vary across settings and overtime trends. Therefore, with the current global push for reduction of new HIV infection of children, robust and updated information is mandatory so as to improve infant feeding practices in the context of HIV. With this view, the current study was aimed at assessing MIF practice and associated factors among HIV-positive women attending ART clinic in Gondar city's governmental health institutions, Northwest Ethiopia, 2017.

.

## 2. Methods

### 2.1. Study Design, Period, Settings, and Population

An institutional-based cross-sectional study was conducted from May 1 to June 30/2017 in public health institutions in Gondar city which is located 750 km northwest of Addis Ababa—the capital city of Ethiopia. According to the 2017/18 population projection, the total population size of the city was estimated to be 338,646 [22]. From the total population, 23.58% were women in the reproductive age group. The city has one comprehensive specialized hospital, eight public health centers, three private maternity specialty clinics, and one private primary hospital.

All HIV-positive women giving birth within 2 years prior to the data collection date and attending antiretroviral therapy (ART) clinics in public health institutions in Gondar city during the study period were enrolled as a study population.

### 2.2. Sample Size Determination and Sampling Technique

The sample size for the study was determined using Open-Epi Version 2 software by considering the following assumptions: prevalence of MIF practice among HIV-positive women—50% (since no recent previous study conducted in the study settings), level of confidence—95%, and margin of error—5%. This resulted in 384 eligible mothers. By adding 10% nonresponse rate, the sample size of 423 was obtained. However, the calculated sample size and the actual number of the study population were nearly equivalent. So, we took all (485) of the HIV-positive mothers who gave birth within 2 years prior to the data collection date and attending ART clinics in Gondar city governmental health institutions during the study period. The purpose of including mothers giving birth within 2 years prior to the data collection date was to obtain sufficient study participants nearly equivalent to the calculated sample size. The readers need to notice that all HIV-positive women within a 2-year postpartum date were just interviewed about their infant feeding practice in the first six months of child age retrospectively.

### 2.3. Variables of the Study

The outcome variable for this study is mixed infant feeding (MIF) practice which was dichotomized as “Yes” coded to be “1” and “No” coded to be 0. Whereas, the explanatory variables included socio-demographic variables, like age of the mother, age of the child, sex of the child, religion, marital status, residence, mother's educational status, educational status of the husband, mother's occupational status, occupational status of the husband, radio/TV, and monthly income, and obstetric-related variables such as parity, antenatal care (ANC), time of ANC initiation, number of ANC visit, place of delivery, mode of delivery, duration of rupture of membrane (ROM), obstetric complication during pregnancy, duration of labor, infant received ARV prophylaxis, infant test result, duration since ART drug initiation, and received counseling about PMTCT at health facility.

### 2.4. Operational or Term Definitions

ART clinics: stand for both ART and PMTCT clinics.

Exclusive breastfeeding (EBF): the infant feeding practice was considered as EBF if an infant was receiving only breast milk without any other liquids or solids, not even water, except for oral rehydration solution or drops or syrups of vitamins, minerals, or medicines [4].

Exclusive replacement feeding (ERF): the feeding practice was considered to be ERF if an infant was not receiving any breast milk with a diet that provides all the nutrients a child needs (i.e., commercial infant formula milk) during the first six months [4].

Mixed infant feeding (MIF): the feeding practice was reported to be MIF if an infant younger than six months of age was given other liquids and/or foods together with breast milk. This could be water, other types of milk, or any type of solid food [4].

### 2.5. Data Collection Tools, Methods, and Procedures

Data were collected by using a pretested and structured questionnaire, which was adapted from related literature [16, 23] through face to face interview. In addition, a checklist was used to extract certain variables via medical chart review. Ten unemployed midwifery graduates were recruited for the data collection process. These included 8 diploma midwives for data collection and 2 BSc midwives for supervision. A one-day training was provided before the actual data collection commencement. Pretest was done on 5% of the sample size. In addition, a necessary revision was made on the tools after pretest for further clarity. Data collectors have been supervised every day, and samples of respondents were reinterviewed at the exit, and the results were then cross-checked. The questionnaires were first prepared in English, translated into Amharic (local) language, and finally back to English. Each data collector checked the questionnaires for completeness before leaving each study participant. Each questionnaire was reviewed daily for completeness and clarity.

One important issue in this study was the potential introduction of recall bias as women up to 2 years after childbirth were included in the study. Therefore, we had executed numerous efforts to minimize such potential recall biases. First, the questionnaire had been well devised in such a way that it could trigger recall ability. Accordingly, it was structured from simple to complex and recent to past in chronological order. It was also translated to local language to enhance optimal communication. Second, strategies of minimizing recall bias were included in the training. Thus, the interviewers were trained in order that they achieved the following measures; assured that each question was clearly understood by the respondents before the responses, informed the local events that might trigger the respondents' recalling capacity, gave participants sufficient time for recalling long term memory, verified with any record or any other potential source of information such as family members, intimate friends and any other persons who were around at the time of event, and used multiple sources of information like crosschecking the documented records and contacting the concerned health care providers for uncertainties in the documents. Meanwhile, all efforts were done to maintain the confidentiality and privacy issues.

### 2.6. Data Processing and Analysis

Data were checked, coded, and entered into EPI INFO version 7. Data were also then exported to SPSS version 20 for analysis. Both descriptive and analytical statistical procedures were done, and results were presented using tables and texts. A binary logistic regression model was used to identify factors associated with MIF practice. Both bivariate and multivariable logistic regression analyses were carried out. Variables with *P* value <0.2 in the bivariate analysis and had no missing value were fitted to the multivariable logistic regression model. Accordingly, the analysis was controlled for the variables with no missed values such as the age of the mother, residence, marital status, mother educational status, mother's occupation, monthly income, radio/TV, place of delivery, antenatal care, gravidity, obstetric complication, and age of the child. Both COR and AOR with the corresponding 95% CI were computed. Finally, a statistically significant association of each variable was declared based on AOR with its 95% CI and *P* value ≤0.05. Model fitness was checked using Hosmer and Lemeshow goodness of a fit test.

## 3. Results

### 3.1. Socio-Demographic Characteristics

A total of 485 women were interviewed yielding a response rate of 100%.

The majority (62.1%) of the participants were in the age group of 25-34 years with the mean ± standard deviation (SD) of 32.2 ± 5.2. More than half (51.5%) of the women were housewives by occupation and considerable numbers (40.9%) of the women had never attended formal education. Large numbers (88.5%) of the mothers were from urban areas. About 251 (51.8%%) of the respondents' families had earned below 36.0 US dollars per month (Table 1).

### 3.2. Obstetric Related Characteristics

A large number (77.3%) of the mothers had ANC visit with the mean ± SD of 3.6 ± 0.8. Nearly one-fifth (19.2%) of the respondents gave birth for the first time. About 366 (75.5%) of the participants had either radio or television. Of 375 mothers who had ANC visit in the most recent pregnancy, almost all (98.7%) of them had received counseling on PMTCT (Table 2).

### 3.3. Proportion and Determinants of MIFP

The proportion of HIV-positive women practicing MIF was found to be 21.6%. Whereas, about 73.8% and 4.5% of the mothers demonstrated EPF and ERF, respectively, in the first six months of infant's age.

Findings from the Bivariate logistic regression analysis showed that mother's educational status (unable to read and write), husband's educational status (unable to read and write), not having ANC visit, late initiation of ANC, home delivery, urban residence, low family monthly income, not owing radio/television, late child age at diagnosis, and infant not given prophylaxis were statistically associated with MIF practice. However, only two variables (i.e., lack of ANC and home delivery) were significantly associated with MIF practice in the final model.

Thus, mothers with no ANC follow-up were 6.9 times more likely to practice MIFP compared with the reference group (AOR = 6.9; 95% CI: 3.4, 14.1). Likewise, the odds of demonstrating MIF were 2.8 times higher among women who underwent home delivery than their counterparts (AOR = 2.8; 95% CI: 1.4, 5.4) (Table 3).

## 4. Discussion

This analysis investigated the proportion and associated factors of MIF practice among HIV-positive women in the study area. The analysis exhibited the high proportion of MIF practice and was significantly associated with lack of ANC visit and home delivery. The proportion of HIV-positive women practicing MIF was found to be 21.6%. The figure is comparable with the findings of the studies done in Addis Ababa—23.3% [24] and a systematic review and meta-analysis finding of Ethiopian pooled prevalence—23.11% [13].

However, it is higher than reports from other studies conducted in Ethiopia such as Debremarkos—14.2% [25], Bahir Dar—10.5% [10] and 16.4% [26], Adama—15.3% [27], Tigray—6.6% [28], and Oromia region—8.3% [29]. It is also larger as compared to findings of the study done in Urban Kano, Nigeria—7.4% [30], Lesotho—10.5% [31], and Gert Sibande, South Africa—12.4% [32]. A number of reasons could be mentioned for the observed higher figure of MIF practice in the current study. First, it might be related to th poor educational status of the participants. The educational status of the participants in the current study is lower than that of the participants involved in the previous studies. For example, about 40.9% of respondents in this study had never attended formal education as compared to only 27.6% in the study done in Tigray. In this perspective, evidences speculate that more educated and aware people are likely to act in accordance with the recommended health care practices [31, 33, 34]. Another possible reason could be associated with a relatively lower proportion of ANC attendees in the current study compared with the previous studies. For instance, a comparatively higher number (91.8%) of the respondents in Tigray had received ANC as compared to that of only 77.3% in the current study and, in fact, adherence to ANC is a preventive independent correlate of MIF practice as it is evidenced in the current multivariable analysis. Equally, the higher proportion of MIF practice in this study might be linked to the place of delivery. The current study revealed that considerable (more than one-fifth) of the participants underwent home delivery which is a higher figure compared with the magnitude in the previous studies. Meantime, home delivery is exhibited to be a risk factor for MIF practice as it is observed even in the current analysis. Similarly, the higher figure of MIF practice in our study could be also ascribed to participants' occupation. Nearly half (48.5%) of the respondents in this study were employees (i.e., were nonhousewife by occupation). Thus, nonhousewife mothers might be forced by their respective employers to resume their regular duty earlier to six months infant's age, since the current modified maternal leave policy in Ethiopia allows mothers to leave from their work for only four months postpartum. This situation in turn could enforce women to commence complimentary infant feeding ahead of the endorsed time by supposing that the breast milk alone would not be sufficient for a long duration of time during working hours. Lastly, the disparity might be connected to the possibility of recall bias secondary to the enrollment of women up to two years postpartum in the current study. Our study did not use the 24-hour recall in determining breastfeeding practice as proposed by WHO [35]. Rather, we included women up to 2 years postpartum and interviewed them about their infant feeding practice during the first six months infant's age retrospectively; hence, this might introduce recall bias.

In contrast, the magnitude of MIF practice in the current analysis is lower than what was reported in studies done in India—29% [36], Rural South Africa—46% [37], and Eastern Uganda—64% [38]. The discrepancy could be explained by variation in time of the study since the herein aforementioned studies had been done ten years back; but, people are becoming more aware regarding best health care practices across time trends.

In this analysis, MIF practice is independently predicted by the lack of ANC visit and home delivery for the indexed childbirth.

Empirical evidences show that receiving ANC is a preventive determinant for enormous adverse health outcomes of child birthing process [12, 39]. The current study is come up with findings in accordance with those existing facts. Hence, mothers who had not received ANC at all in the most recent pregnancy were 6.9 times more likely to practice MIF as compared to those women who had attended at least one ANC visit. The result is consistent with the findings of previous studies done in Ethiopia such as Oromia region [29] and Bahirdar City [26]. This significant association conversely entails that infant feeding counseling (i.e., counseling on PMTCT) during ANC visit has a positive role on the appropriate infant feeding practice as it is observed in the current study that almost all (98.7%) of ANC attendees had received PMTCT counseling. This implication is further supported by existing evidences which show a positive correlation between infant feeding counseling during antenatal care and exclusive infant feeding practice [13, 25, 40].

In many texts, home delivery is cited to be a risk factor for numerous bad obstetric outcomes including vertical MTCT of HIV [19, 23, 41, 42]. A similar finding is observed in the current study. Accordingly, the odds of MIF practice, which is frequently mentioned to be a determinant for poor health outcomes including vertical MTCT of HIV, were 2.8 times higher among respondents having a home delivery history in the recent childbirth than their congruent. It is corroborated by the findings of previous studies done in Nigeria [30]. This significant association could be due to the fact that mothers giving birth at health facilities are likely to have favorable knowledge and/or perception towards recommended infant feeding options secondary to the routine counseling services provided by birth attendants. In this aspect, literature advocates that good knowledge and/or attitude towards recommended infant feeding option has a good outcome in relation to endorsed infant feeding practices [28, 31, 33, 34, 43]. It might be due to the reality that mothers who gave birth at the health facilities tend to receive routine infant feeding counseling as an integral PMTCT strategy. The core implication herein is that mothers giving birth at home are so unlikely to receive appropriate feeding counseling services; consequently, they are assumably to demonstrate MIF.

## 5. Conclusion

The proportion of HIV-positive women with MIF practice was comparable with that of the Ethiopian national level pooled magnitude but higher than what was reported in many other previous studies. The odds of MIF practice were higher among mothers who had no ANC visit and underwent home delivery. The predictors of MIF practice were related to poor adherence to maternal health care service utilization. Hence, stakeholders need to work more on ANC and facility delivery service coverage.

## 6. Limitations

This study did not use the WHO's recommendation of the 24-hour recall in the determination of breastfeeding practice. Thus, recall bias might be introduced since women up to two years postpartum were included in the study. Fortunately, a number of efforts had been undertaken to reduce the bias as it is described in the method section of this manuscript. The authors recommended further studies to be done in a qualitative approach. Authors are also glad to recommend researchers to conduct a community-based study in this concept.

## Figures and Tables

**Table 1:** Socio-demographic and economic characteristics of HIV-positive women in care in public health institutions in Gondar city, Northwest Ethiopia, 2017 (*n* = 485).

Variables	Frequency	Percent
Age of mother(in years)		
<24	19	3.9
25-34	301	62.1
>35	165	34
Age of child(in week)		
8-36	194	40
37-48	80	16.5
>48	211	43.5
Sex of child		
Male	251	51.8
Female	234	48.2
Religion		
Orthodox	387	79.8
Islam	94	19.4
Protestant	4	0.8
Marital status		
Married and living together	307	63.3
Married and living separately	29	6
Divorced	79	16.3
Widowed	57	11.8
Never married	13	2.7
Educational status of mother		
Unable to read and write	170	35.1
Can read and write informally	28	5.8
Attend elementary school	155	31.9
Attend high school and above	132	27.2
Have you ever got marriage		
Yes	472	97.32
No	13	2.68
Educational status of husband (*n* = 472)		
Unable to read and write	78	16.53
Can read and write informally	26	5.51
Educated grade1- 8	160	33.90
Educated high school and above	208	44.06
Occupational status of mother		
House wife	250	51.5
Working in governmental institute	43	8.9
Working in non-governmental institute	192	39.6
Occupational status of husband (*n* = 472)		
Governmental employed	102	21.61
Unemployed	370	78.39
Monthly income(US dollar)		
<36.0	251	51.8
>36.0	234	48.2

**Table 2:** Obstetrics related characteristics of HIV-positive women in care in public health institutions in Gondar city, Northwest Ethiopia, 2017 (*n* = 485).

Variables	Frequency	Percent
Parity		
1-3	386	79.6
4 and above	99	20.4
Time of initiation of ANC (*n* = 375)		
Early/<16 weeks	260	69.3
Late/>16 weeks	115	30.7
Number of ANC visit (*n* = 375)		
2 or 1	118	31.5
>3	257	68.5
Place of delivery		
Home	110	22.7
Health center	132	27.2
Hospital	237	48.9
Private clinic	6	1.2
Mode of delivery		
Spontaneous vaginal delivery(SVD)	399	82.3
SVD with episiotomy	57	11.8
Vaginal instrumental delivery	12	2.5
Cesarean delivery	17	3.5
Know duration of ROM		
Yes	474	97.7
No	11	2.3
Duration of ROM (*n* = 474)		
<4 hrs	394	83.1
≥4 hrs	80	16.9
Obstetric complication during pregnancy		
Yes	106	21.9
No	379	78.1
Duration of labor known		
Yes	478	98.6
No	7	1.4
Duration of labor (*n* = 478)		
≤12 hrs	372	77.8
>12 hrs	106	21.9
Infant received ARV prophylaxis (*n* = 375)		
Yes	285	76
No	90	24
Infant test result		
Nonreactive	374	77.1
Reactive	111	22.9
How long you have received ART drug?		
<4 years	170	35.1
≥4years	315	64.9
Counseled on PMTCT at ANC (*n* = 375)		
Yes	365	97.33
No	10	2.66

**Table 3:** Determinants of mixed infant feeding practice among HIV-positive women in care in public health institutions in Gondar city, Northwest Ethiopia, 2017 (*n* = 485).

Variables	Mixed infant feeding practice		COR with 95% CI	AOR with 95% CI
	Yes	No		
Age of the mother (in year)+				
<35	70	250	1.04 (0.66,1.64)	
≥35	35	130	1	
Residence+				
Rural	20	36	2.25 (1.24,4.08)	1.53 (0.04,3.86)
Urban	85	344	1	1
Marital status+				
Currently not married	37	112	1.30 (0.82,2.06)	
Currently married	68	268	1	
Mother educational status+				
Unable to read and write	51	119	2.07 (1.33,3.22)	0.60 (0.16,2.23)
Able to read and write	54	261	1	1
Husband educational status				
Unable to read and write	29	49	2.52 (1.50,4.21)	NA
Able to read and write	75	319	1	
Mother occupation +				
Housewife	51	199	1.08 (0.56,2.09)	1
Unemployed	40	122	1.38 (0.70,2.74)	1.76 (0.97,3.19)
Governmental employed	14	59	1	2.22 (0.96,5.14)
Husband occupation				
Governmental employed	15	87	1	NA
Unemployed	88	282	1.81 (0.88,2.71)	
Monthly income (US dollar)+				
Equal to or below 36.00	64	187	1.61 (1.04,2.50)	0.87 (0.32,2.36)
Above 36.00	41	193	1	1
Radio/TV+				
Yes	61	305	1	1
No	44	75	2.93 (1.85,4.66)	1.70 (0.94, 3.09)
Place of delivery+				
Home	62	48	9.97 (6.09,16.33)	2.75 (1.39,5.44)
Health facility	43	332	1	1
ANC follow up+				
Yes	37	338	1	1
No	68	42	14.79 (8.86,24.7)	6.90 (3.38,14.08)
Number of ANC visit (*n* = 375)				
1 or 2	16	102	1.76 (0.88,3.52)	NA
3 or above	21	236	1	
Time of initiation of ANC (*n* = 375)				
16 weeks or early	20	240	1	
After 16 weeks	17	98	2.08 (1.05,4.14)	NA
Gravidity+				
Primigravida	18	75	1	
Multigravida	87	305	1.19 (0.67,2.1)	
Obstetric complication+				
Yes	19	87	1	
No	86	293	1.34 (0.77,2.33)	
Age of child at diagnosis (in months)+				
Less than 18	58	332	1	1
Greater than or equal to 18	47	48	5.61 (3.44,9.14)	2.00 (0.98, 3.75)
Infant given prophylaxis (*n* = 375)				
Yes	32	253	1	NA
No	58	32	14.3 (11.33,32.57)	

∗*P* value <0.01; NA: not applicable due to missed values; +: variables for which the analysis was controlled.

## Data Availability

The datasets employed in the current study can be available from the corresponding author upon the reasonable request.

## Abbreviations

AFASS: Affordable, feasible, acceptable, sustainable, safe
AOR: Adjusted odds ratio
COR: Crude odds ratio
ANC: Antenatal care
ART: Antiretroviral therapy
EBF: Exclusive breastfeeding
ERF: Exclusive replacement feeding
MIF: Mixed infant feeding
MTCT: Mother to child transmission
PMTCT: Prevention of mother to child transmission
ROM: Rupture of membrane
SD: Standard deviation
SSA: Sub-Saharan Africa.
